# Functional connectivity network estimation with an inter-similarity prior for mild cognitive impairment classification

**DOI:** 10.18632/aging.103719

**Published:** 2020-09-13

**Authors:** Weikai Li, Xiaowen Xu, Wei Jiang, Peijun Wang, Xin Gao

**Affiliations:** 1 College of Computer Science Technology, Nanjing University of Aeronautics and Astronautics, Nanjing 211106, China; 2 Universal Medical Imaging Diagnostic Center, Shanghai 20030, China; 3 Department of Medical Imaging, Tongji Hospital, Tongji University School of Medicine, Tongji University, Shanghai 20065, China; 4 College of Mathematics and Statistics, Chongqing Jiaotong University, Chongqing 40074, China

**Keywords:** functional connectivity network, functional magnetic resonance imaging, mild cognitive impairment, Pearson’s correlation, partial correlation

## Abstract

Functional connectivity network (FCN) analysis is an effective technique for modeling human brain patterns and diagnosing neurological disorders such as Alzheimer’s disease (AD) and its early stage, Mild Cognitive Impairment. However, accurately estimating biologically meaningful and discriminative FCNs remains challenging due to the poor quality of functional magnetic resonance imaging (fMRI) data and our limited understanding of the human brain. Inspired by the *inter-similarity* nature of FCNs, similar regions of interest tend to share similar connection patterns. Here, we propose a functional brain network modeling scheme by encoding *Inter-similarity* prior into a graph-regularization term, which can be easily solved with an efficient optimization algorithm. To illustrate its effectiveness, we conducted experiments to distinguish Mild Cognitive Impairment from normal controls based on their respective FCNs. Our method outperformed the baseline and state-of-the-art methods by achieving an 88.19% classification accuracy. Furthermore, *post hoc* inspection of the informative features showed that our method yielded more biologically meaningful functional brain connectivity.

## INTRODUCTION

Alzheimer’s disease (AD), a neurodegenerative disorder and the most common cause of dementia [1], seriously interferes with daily life, affecting memory, the ability to reason and communicate, and eventually causing death. According to a recent study [2], the incidence of AD doubles every 5 years after the age 65. Unfortunately, there is still no effective treatment for AD. Therefore, there is ample opportunity to develop pharmacological and behavioral interventions for delaying the onset and progression of AD during its early stages. According to recent statistical analyses [3], ~10-15% of AD patients with mild cognitive impairment (MCI) progress from the prodromal stage of AD to probable AD [4]. Early treatment is believed to delay AD progression at the MCI and preclinical stages [5, 6].

Functional magnetic resonance imaging (fMRI) is a non-invasive technique that can effectively measure brain activity [7–9]. However, it is still challenging to diagnose AD patients using fMRI since spontaneous brain activity can be random and asynchronous across subjects and scanners. By virtue of the brain connectome, the functional brain network provides more consistent data [10–14]. Indeed, functional connectivity network (FCN) has been correlated to some neurological and psychological diseases such as autism spectrum disorder (ASD) [15, 16], MCI [12, 17–19], and AD [20–22], among others, relying heavily on the quality of the final estimated FCNs. Therefore, computing reliable FCNs can increase the accurate diagnosis of such disorders [23].

Mathematically, FCN can be formulated in a graph format, in which each node corresponds to a specific region-of-interest (ROI) in the brain and each edge delineates the relation between the blood-oxygen-level-dependent (BOLD) signals associated with a pair of ROIs. The most widely-used FCN estimation models are based on second-order statistics (or correlations) and, according to a recent review [24], these correlation-based methods are generally more sensitive than complex high-order methods. Therefore, in this paper, we mainly focus on correlation-based methods, and will briefly review some of them, including Pearson’s correlation (PC) [25], sparse representation (SR) [26, 27], and their variants. However, the FCN commonly has more “topological structures” than just sparsity (Sporns 2011). Currently, several studies have proposed more discriminative FCNs with improved estimations to diagnose neurodegenerative diseases. Most of these can be explained under a regularization framework, which illustrates that a reliable FCN estimation model should not only fit the data well, but also effectively encode priors of the brain organization [28]. In practice, the commonly-used priors include sparsity, modularity, group-sparsity, low-rank and scale-free [19, 25, 26, 28, 29], which can be transformed into corresponding regularization terms for FCN estimation. Moreover, the priors can also be transferred from the data modelling [23] or other high-quality data [30]. Such approaches commonly improve the performance of FCNs and their diagnostic accuracy.

In this study, inspired by the fact that similar ROIs in FCNs tend to have similar connection patterns (*i.e.,* inter-similarity structure), we present a novel FCN estimation scheme by encoding such a prior in the form of a graph regularizer. We formulated this prior into a graph-learning model with an additional graph/manifold regularizer for FCN estimation, and further proposed an efficient global optimization algorithm. Additionally, the proposed method is not competing with any other FCN estimation model, since it only provides an effective inter-similarity module in FCN estimation.

## RESULTS

### Network visualization

**Figure 1:**
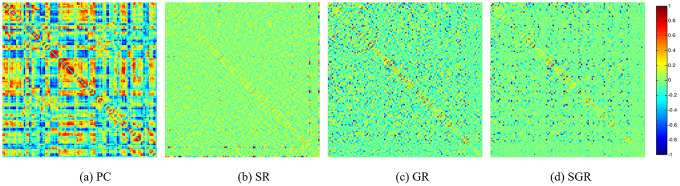
**The FCN adjacency matrices of a certain subject, constructed by different methods.**

For visual comparison of the FCN by PC, SR, GR and SGR methods, we constructed an FCN adjacency matrix **W** for each method (Figure 1), with all weights normalized between −1 and 1, for ease of comparison across the different methods.

Figure 1 shows that the full correlation-based FCNs have different topology from the partial correlation-based FCN (*i.e*., SR, GR and SGR), since they adopt different statistical information by using different data-fitting terms. In addition, compared with SR and GSR, the FCN estimated by SGR tends to be better organized, illustrating the effectiveness of the performance.

### MCI identification

A set of quantitative measurements, including accuracy, sensitivity, specificity, and area under the curve (AUC), are used to evaluate the classification performance of four different methods (PC, SR, GR and SGR). The mathematical definition of the first three measures are as follows:

$$Accuracy=\frac{TruePostive+TrueNegative}{\begin{array}{c} TruePostive+FalsePostive+\\ TrueNegative+FalseNegative \end{array}}$$ (1)

$$Sensitivity=\frac{TruePostive}{TruePostive+FalseNegative}$$ (2)

$$Specificity=\frac{TrueNegative}{TrueNegative+FalsePostive}$$ (3)

Here, *TruePositive* is the number of the positive subjects that are correctly classified in the MCI identification task. Similarly, *TrueNegative*, *FalsePostive* and *FalseNegative* are the numbers of their corresponding subjects, respectively.

**Figure 2:**
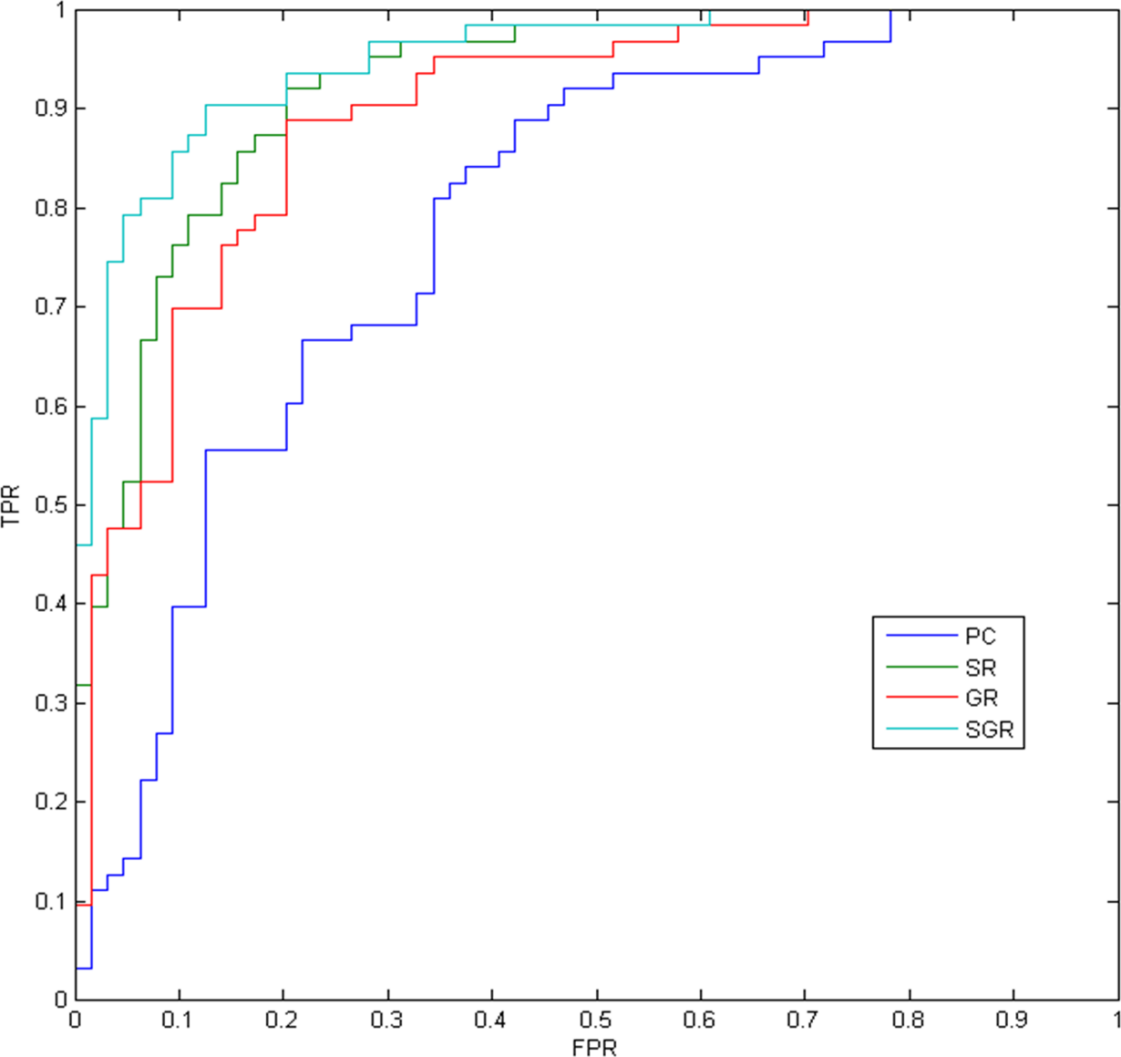
**The ROC results of different methods.**

The MCI vs NC classification results on the ADNI dataset are given in Table 1 and Figure 2, with SGR achieving the best results. As seen in Table 1, the partial correlation-based methods work better than the PC method, which reveals the effectiveness of partial correlation information. In addition, the SGR method strongly outperforms the SR and GR methods, which demonstrates the effectiveness of both sparsity and inter-similarity priors.

**Table 1:** Classification performance corresponding to different FCN estimation. methods on ADNI dataset.

**Method**	**Accuracy**	**Sensitivity**	**Specificity**	**AUC**
PC	71.65	76.56	66.67	0.7852
SR	84.25	85.71	82.81	0.9208
GR	80.31	79.69	80.95	0.8918
SGR	**88.19**	**87.50**	**88.89**	**0.9486**

### Sensitivity to network model parameters

**Figure 3:**
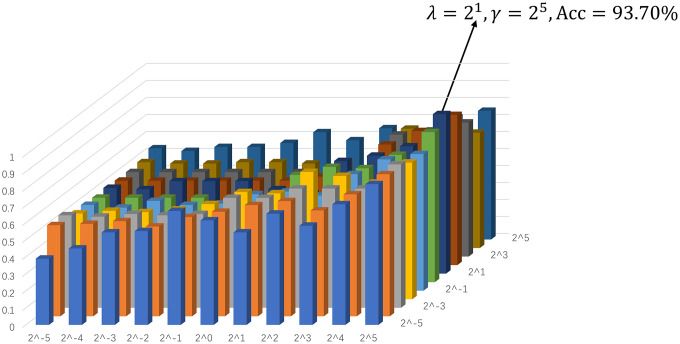
**Assification accuracy based on the networks estimated by the proposed method with different regularized parametric values in the interval [2^−5^, 2^5^].** The results are obtained by LOO test on all subjects.

The ultimate classification accuracy is particularly sensitive to the network model parameters. In Figure 3, we show the classification accuracy corresponding to different parametric combinations in the proposed SGR method. In addition, the classification accuracy is computed by the LOO test on all of the subjects. Consequently, Figure 3 shows that we achieve the best accuracy (93.70%) with λ = 2^1^ (for sparsity) and γ = 2^5^ (for inter-similarity).

### Consensus connections

**Figure 4:**
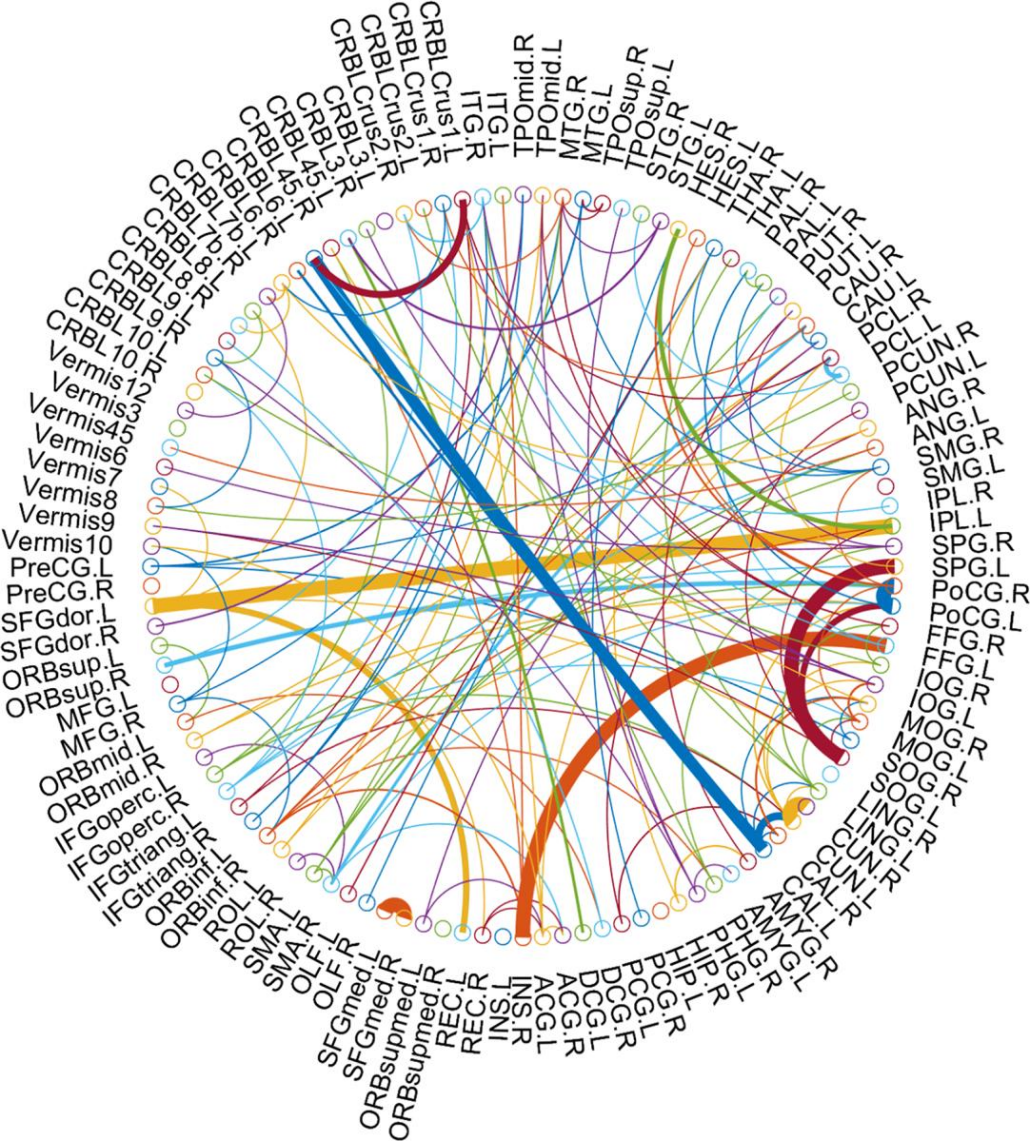
**The consensus connections, selected via LOOCV, between MCI and NC for 116 AAL template ROIs.** The arc thickness indicates the discriminative power of an edge, which is inversely proportional to the estimated p-values. The arc colors were randomly generated to differentiate ROIs. This figure was created using a Matlab function, circularGraph, shared by Paul Kassebaum (http://www.mathworks.com/matlabcentral/fileexchange/48576-circulargraph).

As the selected connections in each inner loop might be different, we recorded the consensus connections for the classification model in each inner LOOCV loop. As mentioned above, we selected the consensus connections with p-value < 0.01 in each loop, and the consensus connections are shown in Table 2 and Figure 4. Specifically, 8 positive consensus connections are listed in Table 2. Most of these discriminative connections were distributed in the frontal, occipital, and parietal lobes. All consensus connections had both enhanced and weakened functional connections in MCI patients. Furthermore, we projected them into the corresponding subnetworks and found that most consensus connections were mainly distributed in the default mode network (DMN), frontoparietal task control network, and sensory/somatomotor hand network.

**Table 2:** Consensus connections between MCI and NC for 116 AAL template ROIs.

**Region**	**Region**	**Mean values**		**P value**
		**MCI**	**NC**	
Frontal_Sup_Medial_L	Frontal_Sup_Medial_R	0.274	0.181	9.72⊆10^-8^
Postcentral_L	Postcentral_R	0.171	0.135	1.14⊆10^-6^
Parietal_Sup_L	Occipital_Sup_L	0.024	0.052	4.49⊆10^-6^
Cuneus_L	Cuneus_R	0.154	0.173	8.89⊆10^-6^
Fusiform_L	Insula_R	0.036	0.007	1.04⊆10^-5^
Cuneus_L	Calcarine_L	0.099	0.048	2.18⊆10^-5^
Cerebelum_Crus1_L	Cerebelum_6_L	0.083	0.118	2.40⊆10^-5^
Occipital_Inf_L	Occipital_Mid_L	0.142	0.114	1.59⊆10^-4^

### Hub regions of functional network

According to the definition of “hubs”, we identified hub nodes of the FCN estimated by SGR with λ = 2^1^ and γ = 2^5^ in MCI patients and NCs. As shown in Table 3, the common hubs of MCI and NCs were located mainly in bilateral middle frontal gyrus, bilateral inferior temporal gyrus, right superior frontal gyrus, right insula and right fusiform gyrus. Most of them were mainly distributed in the DMN, fronto-parietal task control and salience network. Furthermore, it is notable that some hubs were present only in MCI patients and absent in NCs, such as left superior frontal gyrus and left insula. Meanwhile, some hubs were present only in NCs and not in patients with MCI. They were located in the right middle temporal gyrus, left precentral gyrus and left postcentral gyrus. These discriminative brain regions between MCI and NCs were distributed mainly in the DMN, fronto-parietal task control and sensory/somatomotor hand network.

**Table 3:** Hubs in MCI and NCs defined with the degree.

	**AAL Number**	**Corresponding brain regions**	**Sub-networks**
MCI	8	Frontal_Mid_R	Fronto-parietal task control
	56	Fusiform_R	Default mode network
	3	Frontal_Sup_L	Default mode network
	4	Frontal_Sup_R	Default mode network
	7	Frontal_Mid_L	Default mode network
	23	Frontal_Sup_Medial_L	Default mode network
	29	Insula_L	Salience network
	30	Insula_R	Salience network
	89	Temporal_Inf_L	Default mode network
	90	Temporal_Inf_R	Default mode network
NC	7	Frontal_Mid_L	Fronto-parietal task control
	8	Frontal_Mid_R	Fronto-parietal task control
	4	Frontal_Sup_R	Default mode network
	30	Insula_R	Salience network
	86	Temporal_Mid_R	Default mode network
	1	Precentral_L	Sensory/somatomotor Hand
	56	Fusiform_R	Default mode network
	57	Postcentral_L	Sensory/somatomotor Hand
	90	Temporal_Inf_R	Default mode network
	89	Temporal_Inf_L	Default mode network

AAL: the automated anatomical labeling atlas.

### Altered topological properties of functional networks in MCI patients

Based on the FCNs estimated by SGR with λ = 2^1^ and γ = 2^5^, several global graph theory metrics as shown in Table 4, including clustering coefficients (C_p_), shortest path length (L_p_), normalized clustering coefficient (γ), normalized characteristic path length (λ), small-world (σ), global efficiency (E_global_) and modularity (Q), were calculated to elaborate on the topological properties of functional networks in MCI and NC groups. As shown in Table 3, both groups fit γ=C_p_^real^ / C_p_^rand^ > 1, λ=L_p_^real^ / L_p_^rand^ ≈1 and σ=γ/λ > 1. Therefore, FCNs estimated by SGR in MCI patients and NCs showed small-world topological attributes [59]. This means that the brain networks of the two groups maintain an economic and efficient brain network that optimizes the balance between local specialization and global integration [60–62]. Further comparison suggested that the L_p_ values of MCI patients were lower than those in the NC groups (P<0.01), which indicated a reduction of network integration in global information processing in MCI patients. Moreover, the decreased values of γ and Q in MCI patients suggest a reduction of network segregation in local information processing.

**Table 4:** Comparison of topological properties between MCIs and NCs.

	**MCI**	**NC**
C_p_	0.125±0.003	0.147±0.006
L_p_^*^	0.746±0.003	0.759±0.005
γ^*^	1.109±0.071	1.205±0.094
λ	1.040±0.010	1.051±0.015
σ	1.066±0.058	1.149±0.091
E_global_	0.271±0.001	0.278±0.002
Q^*^	6.616±0.638	6.736±1.102

* P<0.01

## DISCUSSION

Here, we proposed a new method to estimate functional brain networks (FCNs) to improve the accuracy of FCN-aided disese diagnosis. To test the effectiveness of our proposed algorithm, we used it to estimate an FCN from experimental fMRI data of AD patients and controls. Estimated FCNs are used to identify MCI patients, which is important for early diagnosis and intervention. Our approach yielded competitive results through three main contributions:

We introduced a graph regularizer into the proposed FCN learning framework for estimating *inter-similarity* FCNs, and combined it with sparse penalty for constructing both *sparse* and *inter-similarity* FCNs, which illustrated that the proposed method scales well.

We used the estimated FCNs to identify MCIs from NCs, and our experimental results showed that the proposed method outperforms state-of-the-art methods. Indeed, it achieved an 88.19% classification accuracy based on a simple feature selection (by means of *t*-tests with a fixed p value) and classification (via linear support vector machines (SVMs) with default parameter C = 1) pipeline.

We explored the selected consensus features (*i.e.*, network connections) in our method and found that most of the selected features tend to be biologically meaningful according to recent studies (Greicius, 2008; Albert et al., 2011), which further illustrated our method’s effectiveness. Moreover, the analysis of graph theory attributes based on our method can be used to further characterize altered patterns and pathological mechanisms underlying the topological properties of brain networks in MCI.

Our simple graph/manifold regularizer was used to estimate an inter-similarity FCNs for each subject. However, FCNs from different subjects tend to share some similar structures [18, 19] and thus the proposed method may lose group information. Therefore, we proposed the development and application of a “group constraint”, such as Group LASSO [63] or tensor low rank [5] to improve FCN computation.

The experiments in our methodological study here constitute a simple verification method for validating the effectiveness of the inter-similarity scheme, without considering other factors (*e.g.*, similarity matrix or classification model). Therefore, we adopted the simplest Pearson Correlation matrix and linear SVM model. Future studies could further improve MCI classification performance.

The distribution of consensus connections and hub nodes indicated that the discriminative features obtained by our proposed method were mainly distributed in the frontal lobe, occipital lobe and parietal lobe of MCI patients. Projecting them into the corresponding subnetworks, we found that most of these brain regions were mainly distributed in the DMN, frontal parietal task control network, and sensory/somatic motor hand network, especially the DMN. Previous studies, such as [64] and [65], have pointed out that DMN facilitated the early diagnosis and prediction of AD. Our results also showed that DMN provided the most discriminating information, which was verified by our proposed method, whose reproducibility we demonstrated here.

The topological properties analyzed in our study suggested that both MCI patients and NCs fitted the small-world attribute in the global topological property. That is, the brain network of MCI and NC groups conform to “economic small-world”, which can provide rapid, real-time information processing across separate brain regions to maximize efficiency with minimal cost, eliciting resilience against pathological attacks [60, 61, 66]. Further comparison suggested that the value of Lp in MCI patients was lower than that in NC groups, which indicated a reduction of network integration in global information processing in the former. Moreover, the decreased values of γ and Q in MCI patients further suggested a reduction of network segregation in local information processing. Therefore, the altered pattern of topological properties obtained by our proposed method indicated a disruption of network integration and segregation of functions in MCI patients, which further demonstrated the pathological mechanisms of FBN.

In summary, the FCN commonly has more topological structures than just sparsity [13, 14]. Due to the limited understanding of the human brain, estimation of the “ideal” FCNs to explore brain pattern or neuro-disease diagnosis is still an active field of research. Here, we focused on the inter-similarity of the FCNs and formulated it into graph regularizer constraints and validated the proposed method on MCI classification. Our results illustrated that additional topological priors can effectively improve diagnosis performance. Our *post-hoc* analyses further showed that more biologically meaningful functional brain connections were obtained by incorporating the inter-similarity prior.

## MATERIALS AND METHODS

### Data acquisition

To test the proposed method, we analyzed publicly-available neuroimaging data from the Alzheimer’s Disease Neuroimaging Initiative (ADNI) database (http://adni.loni.ucla.edu) [31]. ANDI was launched in 2003 by the National Institute on Aging, the National Institute of Biomedical Imaging and Bioengineering, the Food and Drug Administration, private pharmaceutical companies and nonprofit organizations. Initially, the goal of ADNI was to define biomarkers for use in clinical trials and to determine the best way to measure the treatment effects of AD therapeutics.

For this study, we analyzed data for 127 participants, including 63 MCIs and 64 NCs. The scanning parameters included: TR/TE = 3000/30mm, flip angle = 80, imaging matrix=64×64, 48 slices, 140 volumes, and voxel thickness = 3.3mm. SPM8 toolbox (https://www.fil.ion.ucl.ac.uk/spm/software/spm8/) and DPARSFA (version 2.2) [32] were used to preprocess the fMRI data according to the standard, well-established pipeline. The preprocessing pipeline includes removing the first 10 volumes, Slice timing, Realign, Normalize to the MNI space, Spatially smooth, Temporally Detrend, Regression out covariates based on friction 24 and Temporally filtering (0.01-0.08 Hz). For alleviating the head motion effect and artifacts, we followed previously published strategies [33, 34]. We calculated framewise displacement (FD) and excluded subjects with more than 2.5 min (50 frames) data of FD>0.5 from subsequent analyses [35]. Finally, depending on the automated anatomical labeling (AAL) atlas [36], the pre-processed BOLD time series signals were partitioned into 116 ROIs. At last, we put these time series into a data matrix $\text{X}\in {\text{R}}^{137\times 116}$.

### Functional brain network estimation

After obtaining the fMRI data matrix X from the R-fMRI data, the subsequent task is the FCN estimation. The most commonly used FCN estimation methods are those based on correlation, and since they are more sensitive than some complex higher-order methods [14], we focused on the former in this study. For better notation, we first define the data matrix (*i.e.*, BOLD signal matrix), $\text{X}\in R^{T\times N}$ where T is the number of volumes and N is the number of ROIs. The fMRI time series associated with the *i*th ROI is represented by ${\text{x}}_{i}\in R^{T},i=1,\cdots ,N$. In addition, such approach can also be adopted on data of different modality, such as EEG [37, 38].

### Related methods

As the simplest FCN estimation scheme, Pearson’s Correlation (PC)-based FCN estimation methods are widely using to study FCNs [39]. Then, the edge weights of the FCN $W=(W_{ij})\in R^{N\times N}$ can be calculated by PC as follows:

$$W_{ij}=\frac{{\left(x_{i}-{\bar{x}}_{i}\right)}^{T}(x_{j}-{\bar{x}}_{j})}{\sqrt{{\left(x_{i}-{\bar{x}}_{i}\right)}^{T}\left(x_{i}-{\bar{x}}_{i}\right)}\sqrt{{\left(x_{j}-{\bar{x}}_{j}\right)}^{T}(x_{j}-{\bar{x}}_{j})}}$$ (1)

In Eq. (1), $x_{i}-{\bar{x}}_{i}$is a centralized counterpart of *x_i_.* Due to the effect of the noises mixed in the fMRI data, PC always generates dense FCNs. Thus, a threshold is often used to sparsify the PC-based FCNs for filtering out noisy or weak connections.

Compared with PC measures, the full correlation across ROIs, the interaction among multiple ROIs is neglected due to their cofounding effects. In contrast, the partial correlation is proposed by regressing out the confounding effects from other ROIs. However, partial correlation-based methods can be easily ill-posed due to the need to invert the covariance matrix $\text{ Σ}=X^{T}X$. A base solution is to incorporate an *l*_1_-norm regularizer into the partial correlation model [26], which also naturally incorporates the sparsity prior (SR) of FCN. The model of SR is shown as follows:

$$min_{W_{ij}}{\sum_{i=1}^{n}\left\|x_{i}-\sum_{j\neq i}W_{ij}x_{j}\right\|}^{2}+\lambda \sum_{j\neq i}\left|W_{ij}\right|,$$ (2)

the matrix form is proposed as follows:

$$\begin{array}{c} min_{W}{\left\|X-XW\right\|}_{F}^{2}+\lambda \left\|W_{1}\right\|\\ s.t.W_{ii}=0,\forall i=1,\cdots ,n, \end{array}$$ (3)

Note that the *l*_1_-norm regularizer in Eq. (4) below plays a key role in achieving a sparse and stable solution [26].

According to a recent review [1], functional brain network (FBN) estimation methods, from simple to complex, include Pearson’s Correlation (PC), partial correlation [40], regularized partial correlation [41], Bayesian network [42], structural equation modeling [43], and dynamic casual modeling [44]. Each of these methods, in our view, can be considered as a trade-off among biological interpretability, computational efficiency, and statistical robustness. Consequently, we can naturally incorporate a regularized term and statistical information into the objective function for constructing a new platform to estimate FCNs. More specifically, the platform can be formulated using a matrix-regularized learning framework as follows:

$$\min_{W}f\left(X,W\right)+\lambda R\left(W\right),\;\;s.t.\;W\in \Delta ,$$ (4)

where **f**(**X**, **W**) models the statistical information of FCN, and *R*(**W**) is the regularization term for incorporating biological priors of FCN and stabilizing the solutions. In addition, some specific constraints such as symmetry or positive semi-definiteness may be included in Δ for shrinking the search space of **W**, which provides an effective way for obtaining a better FCN. The λ is a hyper-parameter for controling the balance between the first (data-fitting) term and the second (regularization) term.

In fact, many recently-proposed FCN estimation models [45–48] can be unified under this regularized framework with different design of the two terms in Eq. (5) below. The popular data-fitting terms include ${\left\|W-X^{T}X\right\|}_{F}^{2}$ used in Eq. (2) and ${\left\|X-XW\right\|}_{F}^{2}$ used in Eq. (4), while the popular regularization term is *l_1_*-norm [49]. Beyond unifying the existing methods, the regularized framework also provides a platform for developing new FCN estimation methods. In the following section, we will explain our proposed our model based on this framework.

### Our methods

**Figure 5:**
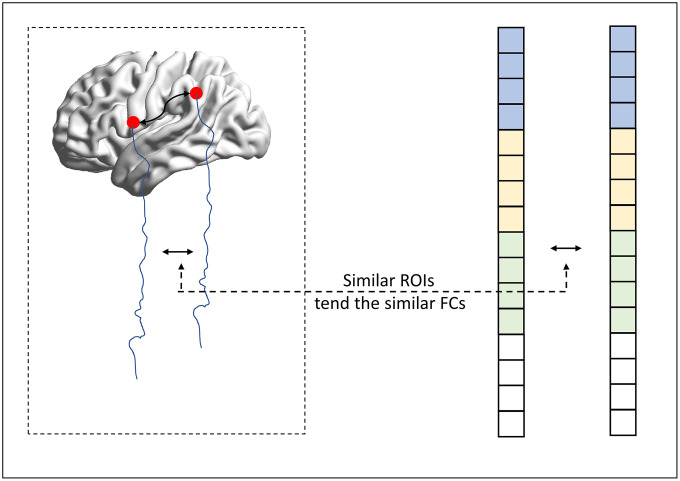
**The motivation of the proposed method.**

As we mentioned above, the regularization-based FCN estimation framework provides an effective scheme for incorporating the biological or physical priors of FCN. In this paper, we try to encode the inter-similarity prior (similar nodes tend to have similar connections) into the FCN estimation. The basic motivation is given in Figure 5.

In particular, we supposed that if two ROIs are defined to be similar, indicating that the connections from these two ROIs should have a similar connection pattern. In this way, we naturally formulate the inter-similarity prior into a graph regularizer (namely MR) penalty, which is given as follows:

$$\begin{array}{c} min_{W}f\left(X,W\right)+\beta \overset{\text{nROI}}{\underset{\text{i},\text{j}=1}{\sum }}{\left\|W_{i,:}-W_{j,:}\right\|}_{2}S_{ij}\\ s.t.W_{ii}=0,\forall i=1,\cdots ,n\; \end{array}$$ (5)

where $S_{ij}$ denotes the similarity between ROI i and ROI j, $W_{i,:}$ represent the weight vector connections from ROI i. Based on the Eq.(5), it is apparent that the more similar ROI i is to ROI j, the estimated $W_{i,:}$ and $W_{j,:}$ will also increase in similarity. To simplify, we can reformulate this into matrix form as follows:

$$\begin{array}{c} min_{W}f\left(X,W\right)+\beta \text{tr(}W^{T}LW\text{)}\text{.}\\ s.t.W_{ii}=0,\forall i=1,\cdots ,n \end{array}$$ (6)

where L is the Laplacian matrix computed as $L=I-D^{-\frac{1}{2}}SD^{-\frac{1}{2}}$, and **D** is a diagonal matrix with each item $D_{ii}=\sum_{j=1}^{nROI}W_{ij}$. The graph S can be defined in many ways such as Pearson’s Correlation, morphological network [50], the network from the high quality data [30] or the predefined graph (must connect or must cannot connect). In particular, in this study, we only consider the positive connection of PC to construct **L**.

Moreover, since the estimated FCN should also be sparse, we further incorporate the *l_1_*-norm penalty into the FCN estimation, and the sparse and graph regularizer (namely **SGR**) is estimated as follows:

$$\begin{array}{c} min_{W}f\left(X,W\right)+\lambda {\left\|W\right\|}_{1}+\beta \text{tr}(W^{\text{T}}LW).\\ s.t.W_{ii}=0,\forall i=1,\cdots ,n \end{array}$$ (7)

In addition, we adopt the partial correlation for the date-fitting term due to its efficiency and effectiveness.

$$\begin{array}{c} min_{W}{\left\|X-XW\right\|}_{F}^{2}+\lambda {\left\|W\right\|}_{1}+\beta \text{tr}(W^{T}LW).\\ s.t.W_{ii}=0,\forall i=1,\cdots ,n \end{array}$$ (8)

For Eq, (8), based on the regularization framework for FCN estimation, we give the optimization algorithm for estimating FCN by SGR. Note that, the objective function of Eq. (8) is convex but indifferentiable due to the $l_{1}$-norm regularizer. A range of algorithms have been proposed for addressing such indifferentiable convex optimization problem in the past few years [51–54]. Here, we select the proximal method [55, 56] to solve Eq. (8) due to its simplicity and efficiency. In particular, for the data-fitting term$f\left(\text{X},\text{W}\right)=\text{X}-{\text{XW}}_{F}^{2}$ (or$\text{W}-{\text{X}}^{T}{\text{X}}_{F}^{2}$) and graph regulaizer term$\text{tr}({\text{W}}^{\text{T}}\text{LW})$, whose gradient w.r.t W is $\nabla_{\text{W}}f\left(\text{X},\text{W}\right)=2{\text{X}}^{T}\text{XW}-{\text{X}}^{T}\text{X}$ (or$\text{W}-{\text{X}}^{T}\text{X}$) and$\nabla_{\text{W}}\text{tr}\left({\text{W}}^{\text{T}}\text{LW}\right)=\text{LW}$. Therefore, we have the following updated formula forW, according to the gradient descent criterion:

$$W_{k}=W_{k-1}-\alpha_{k}(\nabla_{W}f\left(X,W_{k-1}\right)+\beta LW),$$ (9)

where $\alpha_{k}$ denotes the step size of the gradient descent. The initial value of the step size $\alpha_{k}$ will be adaptively updated based on the line search scheme proposed by Nemirovski [57] according to the used SLEP toolbox (http://www.yelab.net/software/SLEP).

Then, for the regularization term $\lambda {\left\|W\right\|}_{1}$ in SGP, the proximal operator for weighted *l*_1_-norm is defined as follows [25]:.

$$\text{pr}(W)={\left[sgn(W_{ij})\times \text{max}(abs(W_{ij})-\lambda ,0)\right]}_{N\times N},$$ (10)

where $sgn(W_{ij})$ and $abs\left(W_{ij}\right)$ return the sign and absolute value of $W_{ij}$, respectively. As a result, two main steps are involved for solving the proposed SGR FCN estimation methods, as given in Table 5.

**Table 5:** Algorithm of SGR-based FCN estimation.

**Input:** X //observed data **Output: W** //functional brain network
**Initialize W**; **while** not converge $W\leftarrow W-\alpha (W-X^{T}X+\beta LW);$ $W\leftarrow proximal_{\lambda {\left\|\;\cdot \;\right\|}_{1}}\left(W\right)={\left[sgn(W_{i,j})\times max(abs\left(W_{i,j}\right)-\lambda ,0)\right]}_{p\times p};$ **end**

### Experimental setting

To validate the proposed FCN method, we conducted experiments on training a classifier for identifying MCI from NCs, based on estimated FCNs. Also, we adopted the SR and PC methods as a baseline for comparison. Since the FCN matrix is symmetric, we used its upper triangular elements as input features for classification. Unfortunately, in our experiment, each FCN had 116 nodes, and thus could produce 6,670 features (corresponding to 6,670 functional connections between 116 ROIs). Compared to the sample size (less than two hundred), the feature dimension was very high, which not only implied expensive computations but would also affect the generalization of the proposed methods. As pointed out in [18], both the feature selection and classifier design have a big influence on accuracy. Thus, in this study, we adopted the simplest feature selection method (*t*-test with p value < 0.01) and the most popular used SVM classifier [58], since our main focus was FCN estimation. In other words, had we not done so, it would have been difficult to conclude whether the FCN construction methods or the feature selection/classification methods contributed to the ultimate performance.

Due to the small sample size, we used the leave one out (LOO) cross-validation strategy to assess the performance of the methods, in which only one subject is left out for testing while the others are used to train the models and get the optimal parameters. To choose optimal parameters, an inner LOO cross-validation was conducted on the training data by grid-search strategy. More specifically, for the regularized parameters  λ and β, the candidate values ranged in $\left[2^{-5},2^{-4},\ldots ,2^{4},2^{5}\right]$; for the hard threshold of PC_threshold_, we used 11 sparsity levels ranging in [1%, 10%,⋯,90%,100%]. For example, 90% means that 10% of the weak edges were filtered out from the FCN. It should be note that selected variables with p-values can be highly complementary to other features, improving the classification result. Thus, to alleviate this issue, the feature selection approach was only applied to the training data.
